# Is less truly more? – reassessing antiretroviral efficacy – a safety analysis for HIV patients switching from triple to double regimens with integrase inhibitors: A systematic review and meta-analysis

**DOI:** 10.1097/MD.0000000000045152

**Published:** 2025-10-17

**Authors:** Francisco Jover, Javier Martínez-Sanz, Antonio Antela, María López-Cavanillas, Minerva Viguera-Moreno, Paloma González-Rodríguez, Pere Domingo

**Affiliations:** a Department of Infectious Diseases, Hospital Clínico Universitario de San Juan, Alicante, Spain; b Department of Infectious Diseases, Hospital Ramón y Cajal, Madrid, Spain; c Department of Infectious Diseases, Hospital Clínico Universitario de Santiago, Santiago de Compostela, Spain; d Medical Affairs Department, Gilead Sciences, Madrid, Spain; e Medical Affairs Department, Outcomes’10 a Product Life-Group Company, Castellón de la Plana, Spain; f Department of Infectious Diseases, Hospital Universitario de la Santa Creu i Sant Pau, Barcelona, Spain.

**Keywords:** antiretroviral therapy simplification, benefits, safety, tolerability

## Abstract

**Background:**

Antiretroviral therapy has marked a transformative advancement in the management of people living with human immunodeficiency virus type-1 (HIV-1) (PLWH), converting this disease into a manageable condition. Triple-drug regimens have long been considered the gold standard for treatment. However, recent developments have focused on 2-drug regimens to mitigate the toxicities associated with polypharmacy while maintaining viral suppression and improving patient outcomes. Although the efficacy of treatment simplification is established, the impact on adverse events (AEs) remains unclear.

**Methods:**

To evaluate the relative risk (RR) of developing drug-related AEs (DRAEs), DRAEs leading to treatment discontinuation (DRAEs-LD), and serious AEs, a systematic review and meta-analysis of available phase 3 and 4 clinical trials lasting at least 48 weeks and assessing treatment simplification to oral INSTIs in virologically suppressed PLWH were conducted. The study also evaluated the effects of early (ES) and late (LS) treatment regimen switches.

**Results:**

Participants who switched to 2DR exhibited a significantly increased RR of developing DRAEs (RR = 6.92; confidence interval [CI]: 3.02–15.86, *P* < .001) and DRAES leading to discontinuation (DRAEs-LD) (RR = 4.41; 95% CI: 1.77–10.99; *P* = .001) compared to those who remained on 3DR/4DR, with no differences observed in the RR of developing serious AEs (RR = 1.06; 95% CI: 0.73–1.55; *P* = .76).

**Conclusion:**

Our findings indicate that there is still limited evidence to confirm that treatment simplification to oral INSTIs improves safety and tolerability profiles in the short-mid term. Our analyses emphasize the importance of evaluating symptom burden when considering therapy regimen switches in clinical practice.

## 1. Introduction

Antiretroviral therapy (ART) has revolutionized the treatment of people living with human immunodeficiency virus type 1 (HIV-1), transforming the disease into a manageable chronic condition. By effectively suppressing viral loads, ART enhances immune function and significantly improves the overall quality of life for those affected.^[1]^ Triple-drug regimens (3DR) have long been the standard of care for treating HIV. These regimens typically combine a modern integrase strand transfer inhibitor (INSTI) with either non-nucleoside reverse transcriptase inhibitors or nucleoside reverse transcriptase inhibitors (NRTIs) as the backbone, effectively maintaining viral suppression and improving patient outcomes.^[2]^ 3DR-based therapies have demonstrated numerous benefits, including reducing morbidity and mortality associated with HIV-1, decreasing transmission rates and restoring baseline levels of cluster of differentiation 4 (CD4+) cell counts, with a lower incidence of complications.^[3]^

However, the chronic nature of the disease and the associated comorbidities require prolonged exposure to multiple antiretroviral drugs, which can lead to cumulative toxicities^[4]^ These long-term effects include reductions in bone density and increased risks for renal, cardiovascular, and metabolic complications, such as diabetes.^[5]^ As a result, efforts are increasingly focused on minimizing these risks while maintaining virological suppression. Over the last decade, 2 drug-based regimens (2DR) have been approved to address these concerns by combining an INSTI with a single transcriptase inhibitor.^[6]^ For this reason, the U.S Department of Health and Human Services (HSS), the European Acquired Immunodeficiency Syndrome (AIDS) Clinical Society, the *Grupo de estudio del SIDA* (GESIDA), and the International Antiviral U.S. Society recommend the 2DR combination of dolutegravir (DTG) with lamivudine (3TC) as initial therapy or a switch option, or rilpivirine (RPV) as a switch option for virologically suppressed patients transitioning from 3DR or a 4-drug regimen (4DR).^[2,7–9]^

These recommendations are based on findings from recent phase III GEMINI-1 and GEMINI-2 studies, which demonstrated that the combination of 3TC + DTG is non-inferior to 3DR regarding efficacy and safety in naïve patients.^[10,11]^ While recent clinical trials confirmed that switching strategy from 3DR or 4DR to 2DR is effective and safe,^[12–15]^ limited evidence has explored the potential impact of this simplification on adverse events (AEs). In this study, we conducted a systematic literature review (SLR) and meta-analysis of available phase III and IV clinical trials evaluating switching strategies from 3-drug (3DR) or 4DR to 2-drug regimens (2DR). We aimed to assess the rates of drug-related AEs (DRAEs), DRAEs leading to treatment discontinuation (DRAEs-LD), and serious AEs while also examining the impact of switching timing (early vs late) on these outcomes. Treatment simplification strategies are not guided by virological criteria but rather by the goal of reducing pharmacological load, improving tolerability, minimizing the associated long-term toxicity, and increasing cost-effectiveness. By compiling evidence, this study aimed to determine if there are benefits of treatment simplification in terms of safety and tolerability to help in the decision-making in routine clinical practice and to provide the best therapeutic option for patients.

## 2. Methods

### 2.1. Systematic literature review

A systematic review phase III and phase IV trials from the last 10 years (2014–2024) reporting DRAEs in virologically suppressed PLWH who switch from 3DR or 4DR to 2DR was carried out following the Preferred Reporting Items for Systematic Reviews and Meta-Analyses (PRISMA) and Cochrane recommendations.^[16,17]^

### 2.2. Data sources and search strategy

The international databases PubMed/Medline, EMBASE, and the Cochrane library, were searched to identify relevant publications for review. Additionally, manual searches were performed to identify conference abstracts presented in the last 2 years (2022–2024) (Annex 1, Supplemental Digital Content, https://links.lww.com/MD/Q301).in the International AIDS Society (IAS/AIDS conferences), the European AIDS Clinical Society, the Conference on Retroviruses and Opportunistic Infections, the IDWeek, and the HIV Drug Therapy Glasgow. The different databases were searched using both MeSH (Medical Subject Headings) and free-text terms, combined with the Boolean connectors “OR” and “AND” (Annex 1, Supplemental Digital Content, https://links.lww.com/MD/Q301).

### 2.3. Study selection

Two independent reviewers performed a 2-level screening of the identified publications. Level 1 entailed a wide screen based on titles and/or abstracts, as available the title and abstract. At level 2, the reviewers independently assessed the full text of the articles, applying the inclusion/exclusion criteria. At both screening levels, discrepancies were resolved by consensus or by involving a third team member (Annex 1, Supplemental Digital Content, https://links.lww.com/MD/Q301).

### 2.4. Eligibility criteria

Publications included Phase III and Phase IV clinical trials from the last 10 years (March 2014 to March 2024) and conference abstracts from the last 2 years (March 2022 to March 2024), published in English, reporting DRAEs, evaluating switch from 3DR or 4DR to oral INSTIs-based 2DR and with a minimum follow-up of 48 weeks for both arms. Publications were excluded if not reporting DRAEs, not published in English, focusing on specific subpopulations (e.g. pediatric), with follow-up periods < 48 weeks, switching to injectable or intramuscular INSTIs, switching to non-INSTIs treatments, or having an inappropriate study design (Phase IV hybrid trials, observational studies, narrative reviews, systematic reviews, meta-analysis, editorial articles, opinion articles or letters to the editor) (Annex 1, Supplemental Digital Content, https://links.lww.com/MD/Q301).

Both phase III and phase IV clinical trials were included in the SLR and meta-analysis to ensure comprehensive coverage of all available evidence on the topic. The included Phase IV trials were not observational, but conducted under controlled clinical conditions, maintaining methodological rigor comparable to Phase III studies. Inclusion of both designs also allowed for sufficient data to perform robust comparisons. Nevertheless, to assess the potential influence of trial phase on the findings, sensitivity analyses were performed excluding phase IV studies to verify the consistency and robustness of the results and conclusions derived from the full dataset.

### 2.5. Data extraction and quality assessment

Data extracted included study design, recruitment period, regimen and number of participants, sociodemographic characteristics (age, sex, race and ethnicity) clinical and treatment characteristics (CD4 + cell count, duration of ART before day 1, type of third agent class) and AE (DRAEs, DRAEs-LD, type of DRAEs and serious AEs). Two independent reviewers extracted all data employing a standardized data extraction form, and discrepancies were resolved by consensus. The quality of included publications was assessed using the quality appraisal checklist recommended by the National Institute for Health and Care Excellence (NICE),^[18]^ with discrepancies being resolved by consensus. The SLR was not publicly registered. Ethical approval was not required for this study, as it is based solely on previously published data from clinical trials (Annex 1, Supplemental Digital Content, https://links.lww.com/MD/Q301).

### 2.6. Meta-analysis

A meta-analysis was performed to determine the relative risk (RR) of developing DRAEs in virologically suppressed PWH who switched from 3 or 4DR to 2DR with oral INSTIs. The RR of developing DRAEs leading to discontinuation and serious AEs were also assessed. Only comparable publications in design (Phase III and Phase IV studies), reported endpoints (comparable DRAEs), and follow-up times (48 weeks) were included in the meta-analysis. The risk of bias (RoB) from the selected studies was determined using the Cochrane RoB tool for randomized trials (RoB 2)^[19]^ (Annex 1, Supplemental Digital Content, https://links.lww.com/MD/Q301).

Heterogeneity among the studies was evaluated employing the heterogeneity index I2 [low (I2 < 25%), moderate (25%<I2 < 50%), high (50%<I2 < 75%), and very high (I2 > 75%)], using STATA software v.14. A random-effects model was applied if heterogeneity was confirmed (I2 > 50% and *P* < .05). Otherwise, a fixed-effects model was employed.^[20]^ Since a single Phase IV study (DOLAM) with a 48-week follow-up was identified from the SLR, and a meta-analysis cannot be performed with data from a single research, data from this Phase IV study were pooled with the 3 Phase III studies. Moreover, additional analyses were conducted excluding the Phase IV study (DOLAM), to evaluate its impact on the overall findings. Lastly, a sensitivity analysis was performed using the alternative method to assess the robustness of the meta-analysis conclusions. Results were reported using a 95% confidence interval (CI), with statistical significance defined as *P* < .05. Data were visually depicted using tables and forest plots. No other variables/outcomes assessment or data conversions were required.

## 3. Results

**Figure 1.**
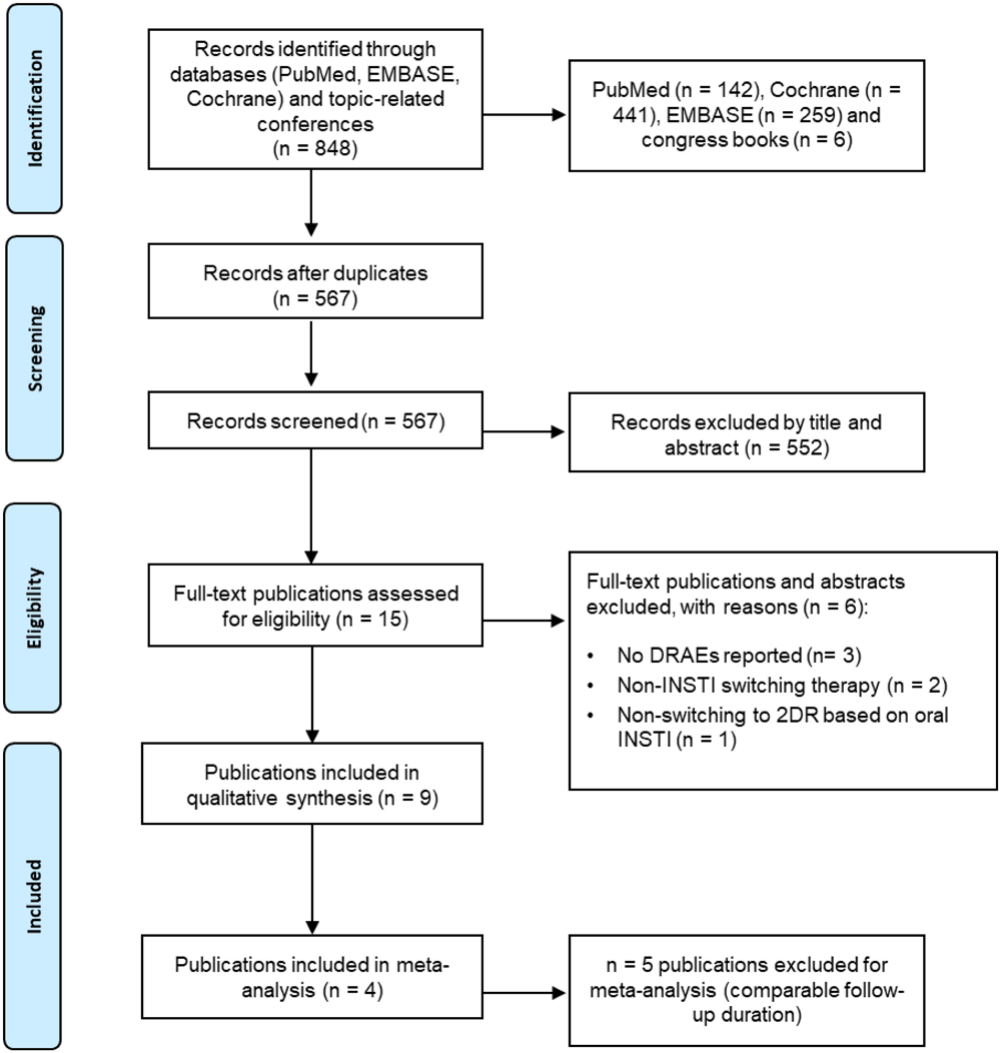
PRISMA flow chart of included publications. PRISMA flow chart illustrating the publications included in the systematic literature review and the meta-analysis. 2DR = 2-drug regimen, DRAE = drug-related adverse event, INSTI = integrase strand transfer inhibitor.

From an initial pool of 848 studies, we ultimately included 9 publications in the SLR and 4 in the meta-analysis (Fig. 1).

The characteristics of the publications included in the SLR are summarized in Table 1. A total of 9 publications were analyzed, encompassing 3 phase III trials: TANGO (three publications), SALSA (one publication) and SWORD (three publications), along with 1 phase IV study, DOLAM (one article). The NICE quality appraisal score analysis indicated a high-quality rating across the selected publications (mean score of 1.67–1.78 out of a maximum of 2). Among them, only 4 publications reported the same follow-up duration (48 weeks), and 3 publications compared early versus late treatment switches. The sample sizes varied across the studies, ranging from 265 to 1024 participants, with well-balanced control and experimental groups in each study. Most participants were male and identified as White, demonstrating stable and comparable CD4 + cell counts (cells/mm³). Additionally, all participants had been on ART for a minimum of 33.8 months.

**Table 1:** Characteristics of the publications included in the systematic literature review and the meta-analysis.

Study- follow-up weeks, study design	Recruitment	Regimen number of participants	Age, median years (range)	Sex (female), n (%)	Race, n (%)	Ethnicity, n (%)	CD4 + cell count mean, n (%) [cells/mm^3^]	Duration of ART before day 1, median of months (range)
TANGO-48, Phase III, randomized, open-label, non-inferiority study	January 18, 2018–May 18, 2018	3DR or 4DR, TAF-BR, 372	39 (18–73)	33 (8.9)	58 (15.6) African American/ African heritage; 13 (3.5) Asian; 289 (77.7) White; 12 (3.2) other	66 (17.7) Hispanic or Latino; 306 (82.3) not Hispanic or Latino	720; 74 (19.9) [<500]; 298 (80.1) [≥500]	35.1 (7.0–160.8)
		2DR, DTG/3TC 369	40 (20–74)	25 (6.8)	50 (13.6) African American/ African heritage; 13 (3.5) Asian; 297 (80.5) White; 9 (2.4) other	69 (18.7) Hispanic or Latino; 300 (81.3) not Hispanic or Latino	682; 98 (26.6) [<500]; 271 (73.4) [≥500]	33.8 (7.1–201.2)
TANGO-96, Phase III, randomized, open-label, non-inferiority study	18th Jan 2018–18th May 2018	3DR or 4DR, TAF-BR, 372	39 (18–73)	33 (8.9)	58 (15.6) African American/ African heritage; 13 (3.5) Asian; 289 (77.7) White; 12 (3.2) other	66 (17.7) Hispanic or Latino; 306 (82.3) not Hispanic or Latino	720; 30 (8) [<350]; 342 (92) [≥350]	35.1 (7.0–160.8)
		2DR, DTG/3TC 369	40 (20–74)	25 (6.8)	50 (13.6) African American/ African heritage; 13 (3.5) Asian; 297 (80.5) White; 9 (2.4) other	69 (18.7) Hispanic or Latino; 300 (81.3) not Hispanic or Latino	682; 35 (9) [<350]; 334 (91) [≥350]	33.8 (7.1–201.2)
TANGO-144, Phase III, randomized, open-label, non-inferiority study	18th Jan 2018–18th May 2018	3DR or 4DR, TAF-BR, 372	39 (18–73)	33 (8.9)	58 (15.6) African American/African heritage; 13 (3.5) Asian; 289 (77.7) White; 12 (3.2) other	66 (17.7) Hispanic or Latino; 306 (82.3) not Hispanic or Latino	720; 74 (19.9) [<500]; 298 (80.1) [≥500]	35.1 (7.0–160.8)
		2DR, DTG/3TC 369	40 (20–74)	25 (6.8)	50 (13.6) African American/ African heritage; 13 (3.5) Asian; 297 (80.5) White; 9 (2.4) other	69 (18.7) Hispanic or Latino; 300 (81.3) not Hispanic or Latino	682; 98 (26.6) [<500]; 271 (73.4) [≥500]	33.8 (7.1–201.2)
TANGO-196, Phase III, randomized, open-label, non-inferiority study	18th Jan 2018–18th May 2018	2DR, DTG/3TC LS 298	43 (20–76)	21 (7)	41 (14) African American/ African heritage; 12 (4) Asian; 235 (79) White; 10 (3) other	NS	751.4; NS	34.0 (7.0–160.8)
		2DR, DTG/3TC ES, 369	40 (20–74)	25 (6.8)	50 (13.6) African American/ African heritage; 13 (3.5) Asian; 297 (80.5) White; 9 (2.4) other	NS	702; NS	33.8 (7.1–201.2)
SALSA-48, Phase III, randomized, open-label, non-inferiority study	11th Nov 2019–23rd Apr 2021	3DR or 4DR, CAR, 247	45 (23–83)	84 (34)	48 (19) African American/ African heritage; 39 (16) Asian; 144 (58) White; 16 (6) other	NS	668; 63 (26) [<500]; 184 (74) [≥500]	71 (12–253)
		2DR, DTG/3TC, 246	45 (22–74)	108 (44)	45 (18) African American/ African heritage; 31 (13) Asian; 149 (61) White; 21 (9) other	NS	675; 60 (24) [<500]; 184 (75) [≥500]	63 (4–240)
SWORD-48, Phase III, randomized, open-label, non-inferiority study	14th Apr 2015–15th Oct 2015	3DR or 4DR, CAR, 511	43 (22–76)	108 (21)	14 (3) American Indian or Alaska native; 50 (10) Asian; 49 (79) African American/ African heritage; 0 native Hawaiian or other pacific islander; 398 (78) White; 2 (<1) other	82 (16) Hispanic or Latino; 429 (84) not Hispanic or Latino	638; NS	*53 (9–270) months*
		2DR, DTG/RPV, 513	43 (21–79)	120 (23)	14 (3) American Indian or Alaska native; 38 (7) Asian; 37 (7) African American/ African heritage; 2 (<1) native Hawaiian or other pacific islander; 421 (82) White; 1 (<1) other	67 (13) Hispanic or Latino; 446 (87) not Hispanic or Latino	611; NS	*51 (8–221*)
SWORD-100, Phase III, randomized, open-label, non-inferiority study	14th Apr 2015–15th Oct 2015	2DR, DTG/RPV LS, 477	43 (22–76)	96 (20)	11 (2) American Indian or Alaska native; 38 (7) Asian; 37 (7) African American/ African heritage; 2 (<1) native Hawaiian or other pacific islander; 421 (82) White; 1 (<1) other	76 (16) Hispanic or Latino; 401 (84) not Hispanic or Latino	611; 315 (93) [≥500]	NS
		2DR, DTG/RPV ES, 513	43 (21–79)	120 (23)	14 (3) American Indian or Alaska native; 38 (7) Asian; 37 (7) African American/ African heritage; 2 (<1) native Hawaiian or other pacific islander; 421 (82) White; 1 (<1) other	67 (13) Hispanic or Latino; 446 (87) not Hispanic or Latino	611; 314 (90) [≥500]	NS
*SWORD-148* Phase III, randomized, open-label, non-inferiority study	14th Apr 2015–15th Oct 2015	2DR, DTG/RPV LS, 477	NS	NS	NS	NS	NS	NS
		2DR, DTG/RPV ES, 513	NS	NS	NS	NS	NS	NS
DOLAM-48, Phase IV, randomized, open-label study	23rd Jun 2015–21st Nov 2019	3DR or 4DR CAR 134	46 (39–51)	18 (13)	NS	25 (19) Latino; 105 (78) White, 4 (3) other	747; NS	NS
		2DR, DTG/3TC 131	45 (37–53)	20 (15)	NS	24 (18) Latino; 106 (81) White, 1 (1) other	700; NS	NS

3TC = lamivudine, ART = antiretroviral therapy, BR = based-regimen, CAR = current antiretroviral regimen, CD4+ = cluster of differentiation 4 positive cells, DR = drug regimen, DTG = dolutegravir, ES = early switch, LS = late switch, NS = not specified, RPV = rilpivirine, TAF = tenofovir alafenamide.

### 3.1. Regimens

The baseline third agent class of participants either continuing with 3DR or 4DR or switching to 2DR was generally consistent across study arms in early-switching trials (Table 2). In the TANGO study, the most common baseline third-agent class used was an INSTI (3DR or 4DR: 79.6%; 2DR: 78.3%), followed by an NNRTI (3DR or 4DR: 12.9%; 2DR: 13.8%) and a protease inhibitor (PI) (3DR or 4DR: 7.5%; 2DR: 7.9%). The SWORD study showed a different trend, with an NNRTI being the most frequent third-agent class (3DR or 4DR: 54 %; DTG/RPV: 50%), followed by a PI (3DR or 4DR: 27 %; 2DR: 26%) and an INSTI (3DR or 4DR: 19 %; 2DR: 20%). In the SALSA study, both treatment arms had an identical distribution, with 50% of participants using an NNRTI as their third-agent class, 40% using an INSTI and 10% using a PI. Similarly, in the DOLAM study, non-nucleoside reverse transcriptase inhibitors were the most common third-agent class (3DR or 4DR: 49 %; 2DR: 51%), with smaller proportions for INSTI (3DR or 4DR: 47 %; 2DR: 54%) and PI (3DR or 4DR: 4 %; 2DR: 5%). The regimens utilized in the late switch studies are outlined in (Table S1, Supplemental Digital Content, https://links.lww.com/MD/Q302).

**Table 2:** Baseline third agent class.

Regimen [n (%)]	TANGO		SALSA		SWORD		DOLAM	
	3DR or 4DR (TAF-BR) (n = 372)	2DR (DTG/3TC) (n = 369)	3DR or 4DR (CAR) (n = 247)	2DR (DTG/3TC) (n = 246)	3DR or 4DR (CAR) (n = 511)	2DR (DTG/RPV) (n = 513)	3DR or 4DR (CAR) (n = 134)	2DR (DTG/3TC) (n = 131)
INSTI	296 (79.6)	289 (78.3)	98 (40)	98 (40)	97 (19)	105 (20)	63 (47)	58 (44)
DTG	NS	NS	41 (17)	45 (18)	NS	NS	27 (20)	21 (16)
EVG/COBI	249 (66.9)	243 (65.9)	27 (11)	24 (10)	NS	NS	23 (17)	25 (19)
BIC	NS	NS	26 (11)	24 (10)	NS	NS	NS	NS
RAL	NS	NS	6 (2)	4 (2)	6 (1)	4 (1)	13 (10)	12 (9)
NNRTI	48 (12.9)	51 (13.8)	124 (50)	123 (50)	278 (54)	275 (54)	65 (49)	67 (51)
RPV	45 (12.1)	43 (11.7)	NS	NS	NS	NS	NS	NS
EFV	NS	NS	73 (30)*	79 (32)*	62 (12)	62 (12)	NS	NS
NRTI	372 (100)†	369 (100)†	NS	NS	NS	NS	NS	NS
ABC/3TC	NS	NS	NS	NS	NS	NS	53 (40)‡	45 (34)‡
3TC	NS	NS	89 (36)*	96 (39)*	NS	NS	--	--
TAF	NS	NS	91 (37)*	83 (34)*	NS	NS	35 (26)‡	38 (29)‡
TDF	NS	NS	109 (44)*	109 (44)*	359 (70)	374 (73)	46 (34)‡	48 (37)‡
FTC	NS	NS	156 (63)*	149 (61)*	341 (67)	352 (69)	--	--
PI	28 (7.5)	29 (7.9)	25 (10)	25 (10)	136 (27)	133 (26)	6 (4)	6 (5)
RTV or COBI -boosted darunavir	27 (7.3)	25 (6.8)	NS	NS	6 (1)	9 (2)	NS	NS

3TC = lamivudine, ABC = abacavir, BIC = bictegravir, BR = based-regimen, CAR = current antiretroviral regimens, COBI = cobicistat, DR = drug regimen, DTG = dolutegravir, EFV = efavirenz, EVG = elvitegravir, FTC = emtricitabine, INSTI = integrase strand transfer inhibitor, NNRTI = nonnucleoside reverse transcriptase inhibitor, NRTI = nucleoside reverse transcriptase inhibitor, NS = not specified, PI = protease inhibitor, RAL = raltegravir, RPV = rilpivirine, RTV = ritonavir, TAF = tenofovir alafenamide, TDF = tenofovir disoproxil fumarate.

* Drug received at screening in ≥30% of participants. N (% of participants with each of the treatments in each arm). Data on individual treatments are incomplete, reflecting the original sources.

† All patients were on a TAF-based regimen prior to randomization.

‡ Backbone regimens (TAF and TDF regimens were combined with FTC).

### 3.2. Adverse events

DRAEs were reported in 7 out of the 9 publications included in the SLR. Across all 7 studies, a higher frequency of DRAEs was consistently observed in participants who switched to a 2DR compared to those who remained on a 3DR or 4DR (12.2 % vs 1.3% in TANGO at 48 weeks, 13.8 % vs 3.2% in TANGO at 96 weeks, 14.9 % vs 4.8% in TANGO at 144 weeks, 19.5 % vs 6.5% in SALSA at 48 weeks, 18.9 % vs 1.7% in SWORD at 48 weeks, and 6.9 % vs 0% in DOLAM at 48 weeks) (Table 3). Additionally, the SWORD study at 100 weeks highlighted a higher incidence of these events in early-switch participants than late-switch participants (20 % vs 12.1%). Similar results were obtained when analyzing DRAEs-LD, with frequencies elevated in participants who switched from 3DR or 4DR to 2DR (2.4 % vs < 1% in TANGO at 48 weeks, 3.8 % vs < 1% in TANGO at 96 weeks, 3.5 % vs 1.3% in TANGO at 144 weeks, 1.6 % vs < 1% in SALSA at 48 weeks, and 2.9 % vs 0% in SWORD at 48 weeks). Nevertheless, SWORD at 100 weeks (3.1 % vs 1.7%) and SWORD-148 (3.5 % vs 2.3%) studies showed a comparable frequency of DRAEs-LD between early-switch participants and late-switch participants. The most frequently reported DRAEs in these studies included headache, diarrhea and insomnia.

**Table 3:** Adverse events.

Study	Regimen	n	DRAE n (%)	DRAE-LD n (%)	Type of DRAE* n (%)										Serious AEs n (%)
					Threshold	Constipation	Depression	Diarrhoea	Flatulence	GFR decreased	Headache	Insomnia	Nausea	Weight Increased	
TANGO-48	3DR or 4DR (TAF-BR)	372	5 (1.3)	1 (<1)	≥0.5%	1 (<1)			0 (0)		0 (0)	0 (0)			16 (4.3)
	2DR (DTG/3TC)	369	45 (12.2)	9 (2.4)	≥0.5%	2 (<1)			2 (<1)		2 (<1)	4 (1)			21 (5.7)
TANGO-96	3DR or 4DR (TAF-BR)	372	12 (3.2)	3 (<1)	≥0.5%	1 (<1)	1 (<1)		0 (0)			0 (0)		1 (<1)	35 (9.4)
	2DR (DTG/3TC)	369	51 (13.8)	14 (3.8)	≥0.5%	2 (<1)	3 (<1)		2 (<1)			4 (1)		2 (<1)	42 (11.4)
TANGO-144	3DR or 4DR (TAF-BR)	372	18 (4.8)	5 (1.3)	≥0.5%	1 (<1)	1 (<1)		0 (0)			0 (0)	2 (<1)	3 (<1)	44 (11.8)
	2DR (DTG/3TC)	369	55 (14.9)	13 (3.5)	≥0.5%	2 (<1)	2 (<1)		2 (<1)			4 (1)	0 (0)	3 (<1)	57 (15.4)
TANGO-196	2DR (DTG/3TC) LS (weeks 148–196)	298	NS	NS											15 (5)
	2DR (DTG/3TC) ES (weeks 1–196)	369	NS	NS											65 (17.6)
SALSA-48	3DR or 4DR (CAR)	247	16 (6.5)	1 (<1)	≥2 participants					0 (0)		1 (<1)		0 (0)	16 (6.5)
	2DR (DTG/3TC)	246	48 (19.5)	4 (1.6)	≥2 participants					2 (<1)		3 (1)		3 (1)	7 (2.8)
SWORD-48	3DR or 4DR (CAR)	511	9 (1.7)	1 (<1)	≥2%			1 (<1)			0 (0)				21 (4.1)
	2DR (DTG + RPV)	513	97 (18.9)	15 (2.9)	≥2%			8 (2)			11 (2)				27 (5.3)
SWORD-100	2DR (DTG + RPV) LS (weeks 52–100)	477	58 (12.1)	8 (1.7)	≥2%			5 (1)			8 (2)		5 (1)		30 (6.3)
	2DR (DTG + RPV) ES (weeks 1–100)	513	103 (20)	16 (3.1)	≥2%			7 (2)			11 (2)		8 (2)		58 (11.3)
SWORD-148	2DR (DTG + RPV) LS (week 52–148)	477	NS	11 (2.3)											44 (9.2)
	2DR (DTG + RPV) ES (weeks 1–148)	513	NS	18 (3.5)											72 (14)
DOLAM-48	3DR or 4DR (CAR)	134	0	NS											6 (4.5)
	2DR (DTG/3TC)	131	9 (6.9)	NS											3 (2.3)

3TC = lamivudine, ABC Abacavir, AEs = adverse events, BIC = bictegravir, CAR = current antiretroviral regimens, COBI = cobicistat, DR = drug regimens, DRAEs = drug-related adverse events, DRAEs-LD = drug-related adverse events leading to discontinuation, DTG = dolutegravir, EFV = efavirenz, ES = early switch, EVG = elvitegravir, FTC = emtricitabine, GFR = glomerular filtration rate, INSTI = integrase strand transfer inhibitor, LS = late switch, M-A = meta-analysis, NNRTI = non nucleoside reverse transcriptase inhibitor, NRTI = nucleoside reverse transcriptase inhibitor, NS = not specified, PI = protease inhibitor, RAL = raltegravir, RPV = rilpivirine, RTV = ritonavir, TAF = tenofovir alafenamide, TDF = tenofovir disoproxil fumarate.

* Type of DRAE occurring in a % of participants in either group.

However, these trends did not persist when assessing the frequency of serious AEs. A heterogeneous response was noted among the studies following the switch to 2DR, with no significant differences observed. Finally, studies comparing early and late switches (TANGO at 196 weeks, SWORD at 100 weeks and SWORD at 148 weeks) reported a greater incidence of serious AEs among participants who underwent early switching (5% vs 17.6%, 6.3% vs 11.3% and 9.2% vs 14% respectively). In these studies, the reported temporal window for evaluating AEs was shorter for late-switch participants compared to early-switch participants [TANGO LS (weeks 148–196) vs ES weeks (1–196 weeks); SWORD LS (weeks 52–100/148) vs ES (weeks 1–148)]. Consequently, when comparing the AEs frequency within the first 48 weeks post-switch for both early-switch and late-switch participants, no differences were observed in TANGO (5.7% vs 5%, respectively) and SWORD (5.3% vs 6.3%, respectively) studies.

### 3.3. Meta-analysis

**Figure 2.**
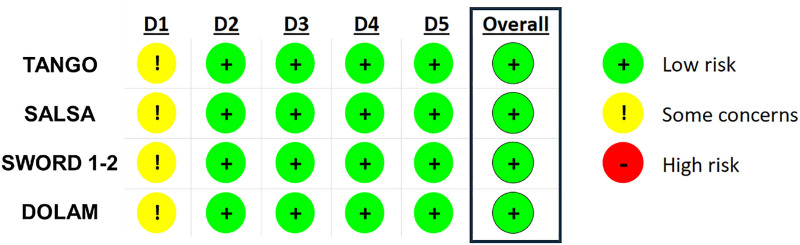
Risk of bias of studies included in the meta-analysis. Figure detailing the risk of bias from the randomization process due to deviated interventions, missing outcome data, measurements of the outcome, or the selection of the reported results of the 4 studies included in the meta-analysis. D1, Bias arising from the randomization process; D2, Bias due to deviations from intended interventions; D3, Bias due to missing outcome data; D4, Bias in measurement of the outcome; D5, Bias in the selection of the reported result.

Phase III studies TANGO, SALSA, SWORD 1 to 2, and Phase IV study DOLAM with a 48-week follow-up duration were included in the meta-analysis. The analysis of the RoB indicated that all studies included had an overall low risk of bias, making them suitable for the analysis (Fig. 2).

### 3.4. Relative risk of developing DRAEs

**Figure 3.**
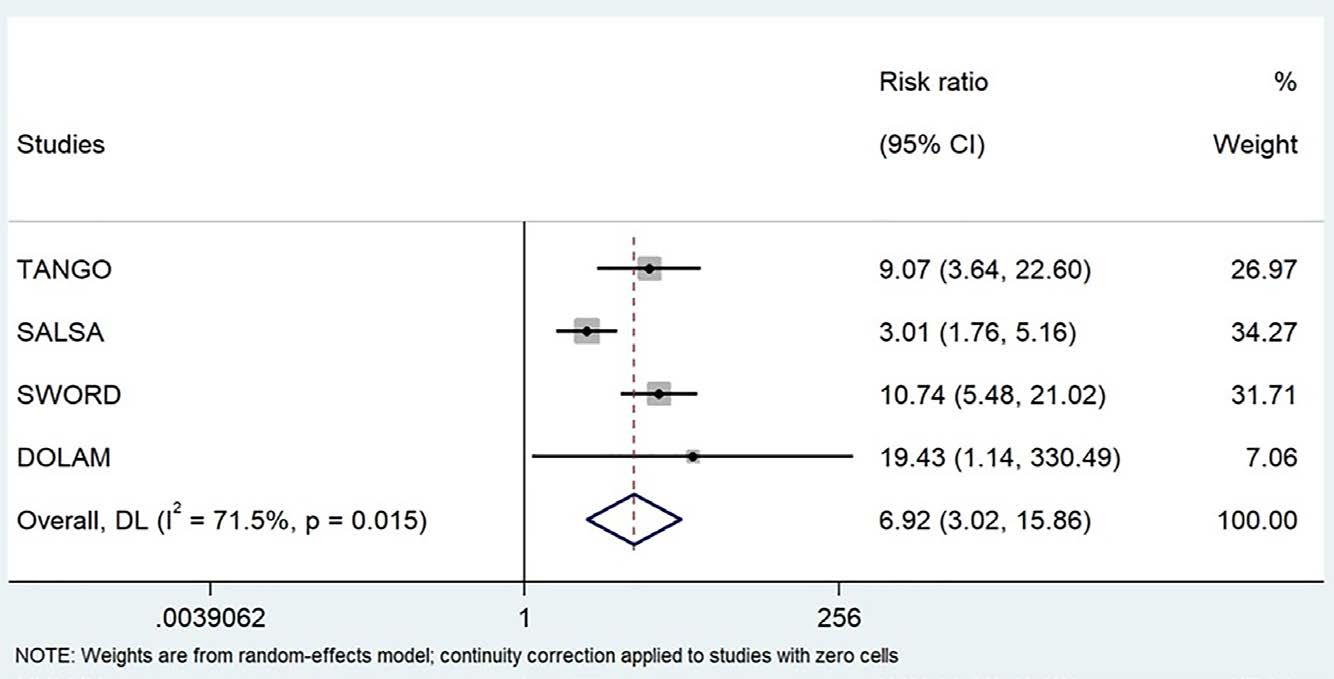
Relative risk of developing DRAEs. Forest plot displaying the risk ratios of developing drug-related adverse events and the relative weights of the 4 studies included in the meta-analysis. Studies included: 4. Participants included: 2523. Continuity correction of 0.5 applied to DOLAM study in zero values. Patients included: 2523. CI = confidence intervals, DRAEs = drug-related adverse events, DL = DerSimonian-Laird estimate of tau², *I*^2^ = heterogeneity index.

Initially, the meta-analysis was conducted to determine the RR of developing DRAEs in virologically suppressed PLWH switching from 4DR or 3DR to a 2DR. A high heterogeneity was observed among the studies (I^2^ = 71.5%, *P* = .015) (Table 4 and Fig. 3). The analysis showed that participants who switched to a 2DR oral INSTIs for 48 weeks presented a significantly higher RR of developing DRAEs compared to those who continued with a 3DR or 4DR (RR = 6.92; CI: 3.02–15.86, *P* < .001). Similar results were observed when only phase III clinical trials were analyzed; heterogeneity (I^2^ = 79.6%, *P* = .008), (RR = 6.41; CI: 2.65–15.64, *P* < .001) (Table 4 and Fig. S1, Supplemental Digital Content, https://links.lww.com/MD/Q303).

**Table 4:** Summary of the meta-analysis results including 48 weeks-follow-up studies.

Outcomes* excluding DOLAM*	No. of studies (name)	No. of participantsexperimental/control group	N^o^ (%) of events experimental/control group	RR	95% Confidence Interval	*P*-value	*I*^2^ %, *P*-value
DRAEs	4 (TANGO, SALSA, SWORD, DOLAM) 3 (TANGO, SALSA, SWORD)	1259/1264*, 1128/1130*	199/30*, 190/30*	6.92*, 6.41*	3.02–15.86*, 2.65–15.64*	<.001*, <.001*	71.5%, .015*, 79.6%, .008*
DRAEs-LD	3 (TANGO, SALSA, SWORD)	1128/1130	28/2	4.41	1.77–10.99	.001	42.3%, .177
Serious AEs	4 (TANGO, SALSA, SWORD, DOLAM) 3 (TANGO, SALSA, SWORD)	1259/1264*, 1128/1130*	28/32*, 25/30*	1.01*, 1.06*	0.7–1.45*, 0.73–1.55*	.973*, .763*	48.8%, .118*, 58.7%, .089*

AEs = adverse events, DRAEs = drug-related adverse events, DRAEs-LD = drug-related adverse events leading to discontinuation, *I*^2^ = heterogeneity index, RR = relative risk.

Values in

* indicate results of an additional analysis excluding the DOLAM study.

### 3.5. Relative risk of developing DRAEs-LD

**Figure 4.**
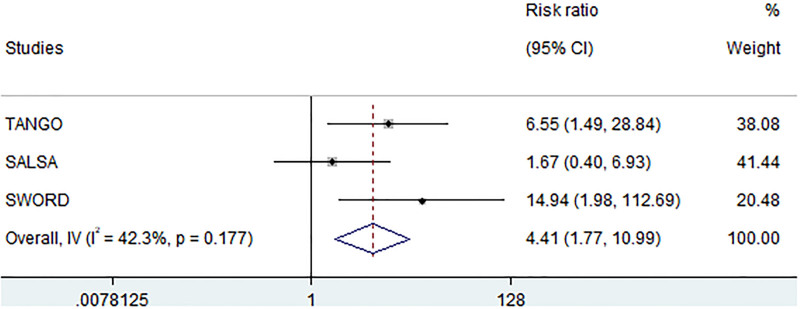
Relative risk of developing DRAEs-LD. Forest plot displaying the risk ratios of developing drug-related adverse events leading to discontinuation and the relative weights of the 3 studies included in the meta-analysis. Studies included: 3. Participants included: 2258. CI = confidence intervals, DRAEs-LD = drug-related adverse events leading to discontinuation, *I*^2^ = heterogeneity index, IV = inverse variance.

A low heterogeneity among the studies (I^2^ = 42.3%, *P* = .177) (Table 4 and Fig. 4). The analysis excluding phase IV trial DOLAM revealed a significantly higher RR of developing DRAEs-LD in those participants performing treatment simplification (RR = 4.41; CI: 1.77–10.99, *P* = .001) (Table 4).

### 3.6. Relative risk of developing serious AEs

**Figure 5.**
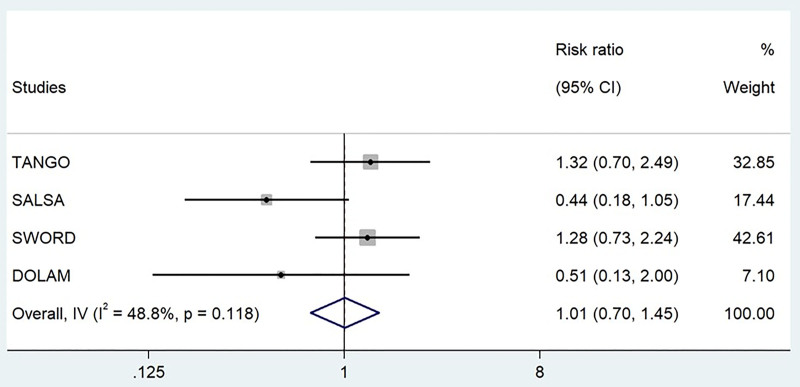
Relative risk of developing serious AEs. Forest plot displaying the risk ratios of developing serious adverse events and the relative weights of the 4 studies included in the meta-analysis. Studies included: 4. Participants included: 2523. AEs = adverse events, CI = confidence intervals, I^2^ = heterogeneity index, IV = inverse variance.

The analysis detected a moderate heterogeneity among the studies (I^2^ = 48.8%, *P* = .118) and a similar RR of developing these serious events when comparing between arms (RR = 1.01; CI: 0.7–1.45, *P* = .973) (Table 4 and Fig. 5). Consistent results were obtained after excluding the phase IV study (heterogeneity, I^2^ = 58.7%, *P* = .089), with no changes in the RR (RR = 1.06; CI: 0.73–1.55, *P* = .763) (Table 4 and Fig. S1, Supplemental Digital Content, https://links.lww.com/MD/Q303).

### 3.7. Sensitivity analyses

The sensitivity analysis yielded similar results to the primary findings (Fig. S2, Supplemental Digital Content, https://links.lww.com/MD/Q303).

## 4. Discussion

Despite a trend towards 2DR for enhanced safety and tolerability in PLWH, the impact of switching from 3DR on these outcomes remains uncertain and warrants further investigation. Current studies suggest that while 2DRs, such as dolutegravir/lamivudine, show good safety profiles, the long-term effects and potential risks associated with switching from established 3DRs require more comprehensive research to fully understand their implications on patient health and treatment efficacy.

Our findings indicate that the switch from continuous 3DR or 4DR to 2DR is not associated with a decreased RR of DRAEs. This conclusion is supported by the findings of the 4 clinical trials analyzed (TANGO, SALSA, SWORD and DOLAM), where DRAEs were consistently higher in 2DR participants than in those on 3DR or 4DR (12.2% vs 1.3%, 19.5% vs 6.5%, 18.9% vs 1.7% y 6.9% vs 0% respectively).^[4,13,15,21]^ During this manuscript preparation, another clinical trial (DYAD) with a similar study design to those included in our meta-analysis also reported an increased frequency of DRAEs among 2DR participants (21% vs 3%).^[22]^ These outcomes further align with those reported in studies involving different formulation strategies. Clinical trials assessing the switch to injectable 2DR therapies (SOLAR and FLAIR) reported higher numbers for DRAEs compared to participants who continued on 3DR or 4DR (72% vs 1% in SOLAR trial and 83% vs 10% in FLAIR trial).^[23,24]^ Although authors suggest that the increased incidence of DRAEs could be linked to the administration route when an injectable is employed, the proportions are still higher in 2DR participants excluding injection-site reactions DRAEs (20% vs 0% in SOLAR trial and 28% vs 10% in FLAIR trial).^[23,24]^

Similar results were obtained when examining the RR of DRAEs-LD. Data from the 3 phase III clinical trials (TANGO, SALSA and SWORD) showed a higher incidence in 2DR participants than those maintaining the 3DR or 4DR (2 vs < 1%, 1.6 vs 1% and 2.9% vs 0%, respectively).^[4,13,21]^ These results align with those reported in the DYAD study (4% vs 0%), and in studies investigating non-oral switching strategies (2% vs 0% in SOLAR trial and 3% vs 1% in FLAIR trial), with authors suggesting that injectable administrations may also play a role in these rates.^[23,24]^ Evidence on the long-term risks of developing DRAEs remains limited, however, some studies gathered in this work included extended follow-up periods.^[12,25]^ Across these studies, DRAEs and DRAEs-LD frequencies were consistently higher in 2DR participants than those in 3DR or 4DR. Importantly, our findings showed no increase in the RR of serious AEs, consistent with previous results^[22]^ and the literature on oral and injectable regimens.^[23,24]^ A recent retrospective study reported higher discontinuation rates of participants switching to 2DR than those who switched to 3DR (19% vs 11%), with toxicity defined as a potential contributing factor.^[26]^

This study analyzed multiple clinical trials with diverse pre- and post-switch treatments, which helps mitigate potential bias associated with the introduction of novel drugs. Notably, prior research has shown a shorter time for discontinuation (*P* = .002) and a higher discontinuation tendency in participants on 2DR, with these individuals showing a 2.3-fold higher likelihood of treatment discontinuation post-switch although reported discontinuations were not due to an increase in AEs.^[27]^ It is worth noting that 2DR have been associated with elevated plasma levels of inflammatory markers, such as interleukin-6, C-reactive protein, and D-Dimer, (*P* = .001, *P* = .003 and *P* = .001, respectively),^[28]^ also reported in clinical trials,^[6,29]^ and 2DR therapy has correlated with glucose and lipid metabolism disruptions, potentially leading to heightened cardiovascular or metabolic risks.^[30]^

Additionally, 2DR have been associated with elevated levels of exhausted lymphocytes (arm–time interaction *P* = .02) and increased monocyte levels (3DR with − 6.7 cells/mm^3^; 95% CI: −16 - +2.6; interaction between arm and time *P* = .03), which account for endothelial affectation.^[31]^ The hypothesis suggested by the authors defines that regimen simplification may impact viral reservoir control, particularly in “sanctuary” organs such as the central nervous system and lymphoid organs, where drug penetration is limited, potentially allowing viral persistence.^[28]^

Overall, the observations reported here differ from another meta-analysis, which concluded that switching does not affect treatment failure, virological failure, DRAEs-LD or the appearance of mutations after 96 weeks.^[32]^ Their analysis, which included 9 studies (some overlapping with those used in our analysis), also incorporated observational studies,^[33,34]^ naïve participants,^[35]^ or non-oral switching strategies.^[24]^ To provide a more precise representation of switching effects in clinical settings, we focused on comparable studies in design, switching strategy, and follow-up duration. Notably, those results align with ours when restricted to clinical trials, as it was reported an elevated RR for DRAEs-LD in participants on 2DR compared to those on 3DR (RR 3.38; CI: 1.58–7.24, *P* = .002).

Recently, Fairhead et al published a letter discussing a similar analysis on switching to 2DR; however, the study with complete data has not yet been published.^[36]^ The conclusions drawn from the available evidence suggest that current research does not confirm that 2DR are safer than 3-drug regimens (3DR). They included trials (SALSA, TANGO, and DOLAM) also analyzed here, assessing DRAEs for up to 48 weeks. Nevertheless, the analysis incorporated studies that were excluded in our study to maintain methodological consistency, since GEMINI-1/2 trials do not assess therapy switching^[10]^ and PASODOBLE trial had a different design.^[37]^

Our findings carry important clinical implications, particularly regarding the current trend of regimen simplification as a default strategy in virologically suppressed PLWH. Healthcare professionals should carefully weigh the potential risks and benefits of switching to simplified strategies on an individual basis, especially in patients with comorbidities, established tolerance to 3DR, or a history of AEs. The incidence of symptoms such as headache, diarrhea, and insomnia reported in the included studies may be clinically relevant, as these symptoms are already prevalent among PLWH.^[38–40]^ Insomnia, in particular, has been linked to increased depressive symptoms, reduced medication adherence, and poorer overall health status, thereby significantly affecting quality of life.^[41]^ Headache affects nearly half of PLWH,^[40]^ and diarrhea occurs at a rate approximately ten times higher than in the general population, with potentially severe consequences in immunocompromised individuals.^[39]^ These symptoms have been shown to cluster in PLWH, further underscoring their cumulative impact on quality of life.^[42]^ Consequently, even modest increases in the frequency or severity of these AEs could meaningfully compromise patient well-being and functional status. Clinicians should assess if, in certain cases, simplification may exacerbate rather than alleviate symptom burden.

Our study has several strengths worth noting. Our study selection provides a broad representation of participants undergoing switching therapy, overcoming some sampling limitations such as the underrepresentation of women and the predominance of White-skin participants.^[4,29]^ Besides, we focused exclusively on clinical trials, minimizing the selection bias often present in observational studies. This study uniquely analyzes DRAEs in an accurately representative clinical context, with comparable populations, follow-up durations, and reported outcomes. Importantly, we focused on participants with prior ART exposure, excluding ART-naïve individuals, to model the switching effects on a population already following these regimens. However, it is also important to consider some limitations. The 48-week follow-up does not allow robust conclusions regarding the benefits in reducing the accumulated toxicity of 2DR switching over extended periods. Besides, participants from the experimental arms in switching studies often stop the regimen when experiencing side-effects, increasing dropout rates. Nevertheless, although different therapeutical combinations were included in the study potentially affecting the observed outcomes, the drugs introduced in the switching strategy were already commercial options. Moreover, some studies evaluating treatment switching have shown improvements in safety and quality of life outcomes.^[43]^ It is also important to acknowledge that the studies included in our analyses did not provide detailed information on the concomitant treatments among their study populations. Therefore, a potential influence of these additional therapies on the observed AEs cannot be ruled out.

## 5. Conclusions

In conclusion, data from phase III and IV clinical trials indicate that therapy simplification does not appear to enhance safety and tolerability profiles at 48 weeks. This study underscores the need for further research to explore the potential safety advantages of 2DR regimens, thereby informing clinical decision-making and optimizing patient care.

## Acknowledgments

The authors would like to thank to Outcomes’10 for the methodological assistance and, specifically Daniel Pinto for their medical writing services, who gives permission to be named. All authors contributed to the study conception, design and participated in the writing and reviewing of the draft versions. All authors approved the final version of the manuscript. This work was supported by Gilead Sciences Spain.

## Author contributions

**Conceptualization:** Francisco Jover, Javier Martínez-Sanz, Antonio Antela, María López-Cavanillas, Minerva Viguera-Moreno, Paloma González-Rodríguez, Pere Domingo.

**Data curation:** Francisco Jover, Javier Martínez-Sanz, Antonio Antela, María López-Cavanillas, Minerva Viguera-Moreno, Paloma González-Rodríguez, Pere Domingo.

**Formal analysis:** Paloma González-Rodríguez.

**Funding acquisition:** María López-Cavanillas, Minerva Viguera-Moreno.

**Investigation:** Francisco Jover, Javier Martínez-Sanz, Antonio Antela, Pere Domingo.

**Methodology:** Francisco Jover, Javier Martínez-Sanz, Antonio Antela, María López-Cavanillas, Minerva Viguera-Moreno, Paloma González-Rodríguez, Pere Domingo.

**Project administration:** María López-Cavanillas, Minerva Viguera-Moreno, Paloma González-Rodríguez.

**Resources:** María López-Cavanillas, Minerva Viguera-Moreno.

**Supervision:** Francisco Jover, Javier Martínez-Sanz, Antonio Antela, María López-Cavanillas, Minerva Viguera-Moreno, Paloma González-Rodríguez, Pere Domingo.

**Validation:** Francisco Jover, Javier Martínez-Sanz, Antonio Antela, Pere Domingo.

**Visualization:** Francisco Jover, Javier Martínez-Sanz, Antonio Antela, María López-Cavanillas, Minerva Viguera-Moreno, Paloma González-Rodríguez, Pere Domingo.

**Writing – original draft:** Francisco Jover, Javier Martínez-Sanz, Antonio Antela, María López-Cavanillas, Minerva Viguera-Moreno, Paloma González-Rodríguez, Pere Domingo.

**Writing – review & editing:** Francisco Jover, Javier Martínez-Sanz, Antonio Antela, María López-Cavanillas, Minerva Viguera-Moreno, Paloma González-Rodríguez, Pere Domingo.

## Supplementary Material






