# Supplementary material for: Is less truly more? – reassessing antiretroviral efficacy – a safety analysis for HIV patients switching from triple to double regimens with integrase inhibitors: A systematic review and meta-analysis
Source: Medicine (Baltimore). 2025 Oct 17;104(42):e45152. doi: 10.1097/MD.0000000000045152 (PMC12537199; doi:10.1097/MD.0000000000045152)
Supplement: Supplementary file 1 [file medi-104-e45152-s001.docx]

**Research Methods**

1. **Systematic Literature Review**

**Eligibility Criteria**

**Inclusion criteria**

- Studies including virologically suppressed PWH
- Studies assessing switching from 3DR to 2DR with oral second-generation INSTIs
- Studies with a minimum follow-up of 48 weeks
- Phase III clinical trials and phase IV studies
- Studies published from 2014 to 2024 (in the last two years for conference abstracts)
- Original articles and conference abstracts
- Studies published in English

**Exclusion criteria**

- Studies including naïve and virologically non-suppressed PWH studies not reporting DRAEs
- Studies in specific population subgroups (e.g. late diagnosed patients, paediatric patients, or patients over 50 years of age)
- Studies assessing switching from 3DR to 2DR with injectable or intramuscular second-generation INSTIs
- Studies assessing switching from 3DR to 2DR with non-INSTI treatments
- Phase IV hybrid trials, observational studies, narrative reviews, systematic reviews, meta-analysis, editorial, opinion articles, and letters to the editor

**Table 1.** Population, Intervention, Comparison, and Outcomes (PICO) framework

| **PICO Framework** | **Inclusion** | **Exclusion** |
| --- | --- | --- |
| P (Population) | Virologically suppressed PWH | - Studies including *naïve and* virologically non-suppressed PWH - Studies in specific population subgroups (e.g. late diagnosed patients, pediatric patients or patients over 50 years of age). |
| I (Intervention) | 2DR switch from 3DR | - Regimens not based on oral second-generation integrase inhibitors |
| C (Comparator) | 3DR | 2DR |
| O (Outcome) | Drug-related adverse events | - |
| S (Study Design) | Phase III clinical trials and phase IV studies | - Phase I and II clinical trials - Follow-up < 48 weeks - Phase IV hybrid trials - Observational studies - Narrative reviews and Meta-analysis |

**Search Strategy**

A systematic review of the literature in international databases was carried out using standardized search filters. The recommendations outlined in the *Preferred Reporting Items for Systematic Reviews and Meta-Analyses* (PRISMA) and Cochrane guidelines were followed.

The different databases were searched using both MeSH (Medical Subject Headings) and free-text terms, combined with the Boolean connectors "OR" and "AND" (Table 2).

**Table 2.** Search terms in databases

| *PUBMED/MEDLINE* | | *COCHRANE LIBRARY* | *EMBASE* |
| --- | --- | --- | --- |
| *Disease* | | | |
| *#1* | *HIV [MesH]* | *HIV* | *HIV .ab,ti.* |
| *#2* | *HIV [tiab]* | *Human immunodeficiency virus* | *Human immunodeficiency virus .ab,ti.* |
| *#3* | *Human immunodeficiency virus [tiab]* | *Adults Living With Human Immunodeficiency Virus* | *Adults Living With Human Immunodeficiency Virus .ab,ti.* |
| *#4* | *Adults Living With Human Immunodeficiency Virus [tiab]* | *PLWH* | *PLWH .ab,ti.* |
| *#5* | *PLWH [tiab]* | *PLH* | *PLH .ab,ti.* |
| *#6* | *PLH [tiab]* | *PWH* | *PWH .ab,ti.* |
| *#7* | *PWH [tiab]* | *Patients with HIV* | *Patients with HIV .ab,ti.* |
| *#8* | *Patients with HIV [tiab]* | *People with HIV* | *People with HIV .ab,ti.* |
| *#9* | *People with HIV [tiab]* | *virologically suppressed* | *virologically suppressed .ab,ti.* |
| *#10* | *virologically suppressed [tiab]* |  |  |
| *Treatment regimen* | | | |
| *#11* | *Treatment switching [MesH]* | *Switching* | *Switching .ab,ti.* |
| *#12* | *Switch* [tiab]* | *third-agent class* | *third-agent class .ab,ti.* |
| *#13* | *third-agent class [tiab]* | *three-drug regimen* | *three-drug regimen .ab,ti.* |
| *#14* | *three-drug regimen [tiab]* | *three-drug combination* | *three-drug combination .ab,ti.* |
| *#15* | *three-drug combination [tiab]* | *triple therapy* | *triple therapy .ab,ti.* |
| *#16* | *triple therapy [tiab]* | *Triple regimen* | *Triple regimen .ab,ti.* |
| *#17* | *Triple regimen [tiab]* | *third-agent class* | *third-agent class .ab,ti.* |
| *#18* | *third-agent class [tiab]* | *two-drug regimen* | *two-drug regimen .ab,ti.* |
| *#19* | *two-drug regimen [tiab]* | *two-drug combination* | *two-drug combination .ab,ti.* |
| *#20* | *two-drug combination [tiab]* | *Dual therapy* | *Dual therapy .ab,ti.* |
| *#21* | *Dual therapy [tiab]* | *Dual regimen* | *Dual regimen .ab,ti.* |
| *#22* | *Dual regimen [tiab]* | *Juluca* | *Juluca .ab,ti.* |
| *#23* | *Juluca [tiab]* | *dolutegravir/rilpivirine* | *dolutegravir/rilpivirine .ab,ti.* |
| *#24* | *dolutegravir/rilpivirine [tiab]* | *Dovato* | *Dovato .ab,ti.* |
| *#25* | *Dovato [tiab]* | *Dolutegravir/Lamivudine* | *Dolutegravir/Lamivudine .ab,ti.* |
| *#26* | *Dolutegravir/Lamivudine [tiab]* | *current antiretroviral regimen* | *current antiretroviral regimen.ab,ti.* |
| *#27* | *current antiretroviral regimen [tiab]* | *CAR* | *CAR .ab,ti.* |
| *#28* | *CAR [tiab]* |  |  |
| *Type of study* | | | |
| *#29* | *Clinical Trials, Phase III as Topic [MesH]* | *Phase III* | *Phase III .ab,ti.* |
| *#30* | *Phase III [tiab]* | *Phase 3* | *Phase 3 .ab,ti.* |
| *#31* | *Phase 3 [tiab]* | *Phase 3a* | *Phase 3a .ab,ti.* |
| *#32* | *Phase 3a [tiab]* | *Phase 3b* | *Phase 3b .ab,ti.* |
| *#33* | *Phase 3b [tiab]* | *Clinical Trials, Phase IV as Topic* | *Phase IV .ab,ti.* |
| *#34* | *Clinical Trials, Phase IV as Topic [MesH]* | *Phase IV* | *Phase 4 .ab,ti.* |
| *#35* | *Phase IV [tiab]* | *Phase 4* |  |
| *#36* | *Phase 4 [tiab]* |  |  |
| *(OR 1-10) AND (OR 11-28) AND (OR 29-36)* | | ***(OR 1-9) AND (OR 11-26) AND (OR 27-33)*** | ***(OR 1-9) AND (OR 10-26) AND (OR 27-32)*** |
| *(("HIV"[MeSH Terms] OR "HIV"[Title/Abstract] OR "human immunodeficiency virus"[Title/Abstract] OR "adults living with human immunodeficiency virus"[Title/Abstract] OR "PLWH"[Title/Abstract] OR "PLH"[Title/Abstract] OR "PWH"[Title/Abstract] OR "patients with HIV"[Title/Abstract] OR "people with HIV"[Title/Abstract] OR "virologically suppressed " [Title/Abstract]) AND ("treatment switching"[MeSH Terms] OR "Switch*"[Title/Abstract] OR "third-agent class"[Title/Abstract] OR "three drug regimen"[Title/Abstract] OR "three-drug combination"[Title/Abstract] OR "triple therapy"[Title/Abstract] OR "triple regimen"[Title/Abstract] OR "third-agent class” OR "two-drug regimen"[Title/Abstract] OR "two-drug combination"[Title/Abstract] OR "Dual therapy"[Title/Abstract] OR "Dual regimen"[Title/Abstract] OR “Juluca” [Title/Abstract] OR “dolutegravir/rilpivirine” [Title/Abstract] OR “Dovato” [Title/Abstract] OR “Dolutegravir/Lamivudine” [Title/Abstract] OR “current antiretroviral regimen” [Title/Abstract] OR “CAR” [Title/Abstract])) AND ("clinical trials, phase iii as topic"[MeSH Terms] OR "phase iii"[Title/Abstract] OR "phase 3"[Title/Abstract] OR "phase 3a"[Title/Abstract] OR "phase 3b"[Title/Abstract] OR "clinical trials, phase iv as topic"[MeSH Terms] OR "phase iv"[Title/Abstract] OR "phase 4"[Title/Abstract]))* | | *(("HIV"OR "human immunodeficiency virus" OR "adults living with human immunodeficiency virus" OR "PLWH" OR "PLH" OR "PWH" OR "patients with HIV" OR "people with HIV" OR "virologically suppressed") AND ("Switching" OR "third-agent class" OR "three drug regimen" OR "three-drug combination" OR "triple therapy" OR "triple regimen" OR "third-agent class” OR "two-drug regimen" OR "two-drug combination" OR "Dual therapy" OR "Dual regimen" OR “Juluca” OR “dolutegravir/rilpivirine” OR “Dovato” OR “Dolutegravir/Lamivudine” OR “current antiretroviral regimen” OR “CAR”) AND ("phase iii" OR "phase 3" OR "phase 3a" OR "phase 3bOR "phase iv" OR "phase 4"))* |  |

**Database Search**

The international databases **PubMed/Medline, ClinicalTrials.gov, EU Clinical Trials Register, Cochrane** and **Embase** were searched to identify relevant studies for review.

**Additional Targeted Search**

Additional searches were performed to identify conference abstract such as the International AIDS Society (IAS/AIDS conferences), the European AIDS Clinical Society (EACS), the Conference on Retroviruses and Opportunistic Infections (CROI), the IDWeek, and the HIV Drug Therapy Glasgow.

**Selection of Studies**

Two independent reviewers screened all identified publications at two levels: Level 1 screening entailed a wide screen based on item titles and/or abstracts, as available. The full-text of all articles passing Level 1 screening were retrieved for Level 2 screening: an ascertainment of final eligibility for the review. Discrepancies were resolved by consensus or by involving a third team member. All screening was recorded using a priori developed inclusion criteria as described above. The reasons for the exclusions of full-text reviewed articles are described in the PRISMA chart.

**Data Extraction Process**

All data were extracted by two independent reviewers. Discrepancies were resolved by consensus or by involving a third team member. Data extractors were not blinded to any study information. Before data extraction began, a standardized data extraction form/database and data extraction guidelines were used upon achieving consensus by the study team on all included data fields.

**Variables**

**Table 3.** Variables extracted from the studies included in de SLR

| Item | Description | Data type |
| --- | --- | --- |
| Title and reference | Title and reference of the study | - Title of the publication |
| Author and publication date | First author and publication date | - First author and publication date |
| Study objective | Objective of the study | - Main objective |
| Study design | Type of study and follow-up time | - Design (open label, single blind, double blind) / (Multicenter, single center) / (randomized, nonrandomized) / (withdrawal trial, parallel group trial, cross-over trial, phase III, phase IV) - Follow-up time |
| Statistical analysis | Type of analysis performed | - Statistical tests |
| Type of treatment | Prior treatment (3DR) [comparator] and treatment after switching (2DR) [intervention] | - Previous and current treatments, n (%) |
| Population | Type of patients, sample size, and main sociodemographic and clinic characteristics* | - Sample Size, n - Baseline third agent class, n (%) - Regimen - Regimen duration, mean (SD) or median (IQR) - Age, n (%) - Sex, n (%) - Race, n (%) - Ethnicity, n (%) CD4 cell category, cells/μL, n (%) - Comorbidities |
| Main end-points | Before and after switching, the following variables are extracted:  DRAE  DRAE-LD  Type of DRAE  AES (≥ 5%)  Serious AE | - DRAE-LD, n (%) - DRAE, n (%) - Type of DRAE, n (%) - AES (≥ 5%), n (%) - Serious AE, n (%) - Laboratory abnormalities [such as AST, ALT, total cholesterol, HDL, and LDL elevation, total cholesterol/HDL ratio, hyperglycemia or creatine kinase (n, %, mean (SD), median (IQR) - Discontinuation due to weight increase up to overweight/obesity levels |
| Location | Location of study | Country |
| Recruitment period | Start and end of recruitment. | Dates, weeks |
| Funding source | Identification of the study funder | Funding source |
| Risk of bias (Rob 2) | Assessment of the risk of bias | - Low, high, or unclear risk of bias (5 domains) |
| Limitations | Main limitations of the study | - Summary of the limitations of the study |
| Methodological quality (NICE) | Assessment of the quality of the included studies | - Quality grading for internal/external validity |

**Missing Data**

Possible missing data was properly identified in the database (e.g. 'NA'). Any missing, unknown, or omitted data was treated as missing data and explained in the final results report.

**Dealing With Duplicate and Companion Publications**

If duplicate publications were identified, priority was given to:

1. Manuscripts over congress posters and abstracts.
2. Posters over congress abstracts
3. **Meta-analysis**

A meta-analysis was carried out to assess each study objective in publications included in the SLR that are similar in:

- Design: Phase III and Phase IV studies were analyzed separately.
- Quality: Risk of bias.
- Participant characteristics: age, gender, ethnicity, baseline third agent class, regimen duration, CD4 cell baseline category, HIV-1RNA baseline level.
- Endpoints reported: Studies that report all necessary data to include in each meta-analysis were considered.

Studies included in the SRL reported AEs outcomes and laboratory parameters. However, not all studies reported all study variables and, in some cases, variables were not comparable across studies. Some examples are the variable “number of AEs in ≥ 5% of participants” (which was not similarly reported between studies, since some studies reported AEs in ≥ 10% of participants), laboratory parameters (expressed in different units between studies and, in some cases, subject to baseline adjustments, making them not comparable across studies) or unreported data in some studies. Moreover, the follow-up times differed between studies. Only four of the nine publications included had the same follow-up period (48 weeks), corresponding to the Phase III clinical trials TANGO, SALSA and SWORD 1-2, and Phase IV study DOLAM. All these four publications similarly reported DRAEs and serious AEs outcomes. Therefore, a meta-analysis was carried out on these publications.

- 1. **Quantitative Synthesis**

The direct meta-analysis technique was applied to compare the proportion of DRAE, DRE leading to discontinuation, and serious AE extracted from SLR in virologically suppressed PWH on 3DR and those who have switched from 3DR to 2DR with oral second-generation INSTIs at week 48. This technique combines the studies’ endpoints to obtain a single measure of the effects of the treatments evaluated. With a very small number of studies, the fixed-effects model may be more suitable option, as the random-effects model requires a precise estimation of heterogeneity. A detailed similarity assessment was performed before the meta-analysis, as described in section 4.2.3. However, if there was evidence of significant heterogeneity, the random-effects model was considered, as it allows for the extrapolation of results by incorporating heterogeneity among studies. *.* Therefore, the following analysis plan was proposed:

1. Heterogeneity assessment. Heterogeneity was evaluated using the I^2^ estimator (although the precision of this estimator may be low due to the small number of studies, it can provide a general idea of the heterogeneity).
2. If heterogeneity was confirmed, the random-effects model was used; otherwise, the fixed-effects model was employed.

Additionally, a sensitivity analysis was conducted using the model not initially chosen. This enabled a comparison of results to evaluate the robustness of the meta-analysis conclusions.

For the fixed-effects model, the inverse variance weighting method was employed. This method allows a higher weight to be assigned to studies with smaller standard errors, thus giving studies with higher precision a greater influence on the results. On the other hand, for the random-effects model, the DerSimonian and Laird weighting method was used. This method individually adjusts the weights of the studies to account for both within-study and between-study standard errors, making it suitable when there is evidence of heterogeneity among the selected or included studies.

The results of the meta-analysis are represented in a forest plot. Endpoints extracted from the SLR were reported as relative risk of patients to develop DRAEs, DRAEs-LD, and serious AEs after switching from 3DR or 4DR to 2DR (using a 95% confidence interval).

- 1. **Assessment of Similarity**

The similarity assessment was performed before the meta-analysis to detect any imbalances across studies that could introduce bias in the results. This assessment consisted of visually inspecting variables related to baseline characteristics.

- 1. **Assessment of Heterogeneity**

Heterogeneity was assessed by the heterogeneity index I^2^, whose values indicate different levels of heterogeneity: low (I^2^<25%), moderate (25%<I^2^<50%), high (50%<I^2^<75%), and very high (I^2^>75%). When obtaining an index I^2^>75% from the analysis, we attempted to determine potential reasons for it by examining individual studies.

- 1. **Other analysis**

Only one Phase IV study (DOLAM) with a 48-week follow-up was obtained from the SLR, so meta-analysis cannot be performed with only one study. We proposed an additional analysis pooling the 4 studies extracted from the SLR (Phase III [n=3] and IV [n=1]).

Four studies were proposed to conduct the meta-analysis. In this regard, when the number of studies in a meta-analysis is very small, the capacity to perform adjustments may be limited. With a small number of studies, subgroup analyses and meta-regressions will be restricted. However, in such cases, conducting sensitivity analyses might be the most appropriate approach.

1. **Assessment of the body evidence**

The body of evidence in the studies included in the SLR was assessed following the quality appraisal checklist (quantitative intervention studies) (Table 4) recommended by the National Institute for Health and Care Excellence (NICE).

Each question in the checklist covers a methodology aspect that significantly affects a study's conclusions. Checklist items are worded so that 1 of 5 responses is possible: 1) ++, Indicates that for that particular aspect of study design, the study has been designed or conducted in such a way as to minimize the risk of bias; 2) +, Indicates that either the answer to the checklist question is not clear from the way the study is reported, or that the study may not have addressed all potential sources of bias for that particular aspect of study design; 3) −, Should be reserved for those aspects of the study design in which significant sources of bias may persist; 4) Not reported (NR), Should be reserved for those aspects in which the study under review fails to report how they have (or might have) been considered; and 6) Not applicable (NA), Should be reserved for those study design aspects that are not applicable given the study design under review (for example, allocation concealment would not be applicable for case control studies).

**Table 4.** NICE quality appraisal checklist

| **Study identification:**(Include full citation details) |  | |
| --- | --- | --- |
| **Study design:**  Refer to the glossary of study designs ([appendix D](https://www.nice.org.uk/article/pmg4/chapter/appendix-d-glossary-of-study-designs)) and the algorithm for classifying experimental and observational study designs ([appendix E](https://www.nice.org.uk/article/pmg4/chapter/appendix-e-algorithm-for-classifying-quantitative-experimental-and-observational-study-designs)) to best describe the paper's underpinning study design |  | |
| **Guidance topic:** |  | |
| **Assessed by:** |  | |
| **Section 1: Population** | | |
| **1.1 Is the source population or source area well described?**  Was the country (e.g. developed or non-developed, type of healthcare system), setting (primary schools, community centres etc.), location (urban, rural), population demographics etc. adequately described? | ++  +  −  NR  NA | Comments: |
| **1.2 Is the eligible population or area representative of the source population or area?**  Was the recruitment of individuals, clusters or areas well defined (e.g. advertisement, birth register)?  Was the eligible population representative of the source? Were important groups under-represented? | ++  +  −  NR  NA | Comments: |
| **1.3 Do the selected participants or areas represent the eligible population or area?**  Was the method of selection of participants from the eligible population well described?  What % of selected individuals or clusters agreed to participate? Were there any sources of bias?  Were the inclusion or exclusion criteria explicit and appropriate? | ++  +  −  NR  NA | Comments: |
| **Section 2: Method of allocation to intervention (or comparison)** | | |
| **2.1 Allocation to intervention (or comparison). How was selection bias minimised?**  Was allocation to exposure and comparison randomised? Was it truly random ++ or pseudo-randomised + (e.g. consecutive admissions)?  If not randomised, was significant confounding likely (−) or not (+)?  If a cross-over, was order of intervention randomised? | ++  +  −  NR  NA | Comments: |
| **2.2 Were interventions (and comparisons) well described and appropriate?**  Were interventions and comparisons described in sufficient detail (i.e. enough for study to be replicated)?  Was comparisons appropriate (e.g. usual practice rather than no intervention)? | ++  +  -−  NR  NA | Comments: |
| **2.3 Was the allocation concealed?**  Could the person(s) determining allocation of participants or clusters to intervention or comparison groups have influenced the allocation?  Adequate allocation concealment (++) would include centralised allocation or computerised allocation systems. | ++  +  −  NR  NA | Comments: |
| **2.4 Were participants or investigators blind to exposure and comparison?**  Were participants **and** investigators – those delivering or assessing the intervention kept blind to intervention allocation? (Triple or double blinding score ++)  If lack of blinding is likely to cause important bias, score −. | ++  +  −  NR  NA | Comments: |
| **2.5 Was the exposure to the intervention and comparison adequate?**  Is reduced exposure to intervention or control related to the intervention (e.g. adverse effects leading to reduced compliance) or fidelity of implementation (e.g. reduced adherence to protocol)?  Was lack of exposure sufficient to cause important bias? | ++  +  −  NR  NA | Comments: |
| **2.6 Was contamination acceptably low?**  Did any in the comparison group receive the intervention or vice versa?  If so, was it sufficient to cause important bias?  If a cross-over trial, was there a sufficient wash-out period between interventions? | ++  +  −  NR  NA | Comments: |
| **2.7 Were other interventions similar in both groups?**  Did either group receive additional interventions or have services provided in a different manner?  Were the groups treated equally by researchers or other professionals?  Was this sufficient to cause important bias? | ++  +  −  NR  NA | Comments: |
| **2.8 Were all participants accounted for at study conclusion?**  Were those lost-to-follow-up (i.e. dropped or lost pre-,during or post-intervention) acceptably low (i.e. typically <20%)?  Did the proportion dropped differ by group? For example, were drop-outs related to the adverse effects of the intervention? | ++  +  −  NR  NA | Comments: |
| **2.9 Did the setting reflect usual UK practice?**  Did the setting in which the intervention or comparison was delivered differ significantly from usual practice in the UK? For example, did participants receive intervention (or comparison) condition in a hospital rather than a community-based setting? | ++  +  −  NR  NA | Comments: |
| **2.10 Did the intervention or control comparison reflect usual UK practice?**  Did the intervention or comparison differ significantly from usual practice in the UK? For example, did participants receive intervention (or comparison) delivered by specialists rather than GPs? Were participants monitored more closely? | ++  +  −  NR  NA | Comments: |
| **Section 3: Outcomes** | | |
| **3.1 Were outcome measures reliable?**  Were outcome measures subjective or objective (e.g. biochemically validated nicotine levels ++ vs self-reported smoking −)?  How reliable were outcome measures (e.g. inter- or intra-rater reliability scores)?  Was there any indication that measures had been validated (e.g. validated against a gold standard measure or assessed for content validity)? | ++  +  −  NR  NA | Comments: |
| **3.2 Were all outcome measurements complete?**  Were all or most study participants who met the defined study outcome definitions likely to have been identified? | ++  +  −  NR  NA | Comments: |
| **3.3 Were all important outcomes assessed?**  Were all important benefits and harms assessed?  Was it possible to determine the overall balance of benefits and harms of the intervention versus comparison? | ++  +  −  NR  NA | Comments: |
| **3.4 Were outcomes relevant?**  Where surrogate outcome measures were used, did they measure what they set out to measure? (e.g. a study to assess impact on physical activity assesses gym membership – a potentially objective outcome measure – but is it a reliable predictor of physical activity?) | ++  +  NR  NA | Comments: |
| **3.5 Were there similar follow-up times in exposure and comparison groups?**  If groups are followed for different lengths of time, then more events are likely to occur in the group followed-up for longer distorting the comparison.  Analyses can be adjusted to allow for differences in length of follow-up (e.g. using person-years). | ++  +  NR  NA | Comments: |
| **3.6 Was follow-up time meaningful?**  Was follow-up long enough to assess long-term benefits or harms?  Was it too long, e.g. participants lost to follow-up? | ++  +  −  NR  NA | Comments: |
| **Section 4: Analyses** | | |
| **4.1 Were exposure and comparison groups similar at baseline? If not, were these adjusted?**  Were there any differences between groups in important confounders at baseline?  If so, were these adjusted for in the analyses (e.g. multivariate analyses or stratification).  Were there likely to be any residual differences of relevance? | ++  +  −  NR  NA | Comments: |
| **4.2 Was intention to treat (ITT) analysis conducted?**  Were all participants (including those that dropped out or did not fully complete the intervention course) analysed in the groups (i.e. intervention or comparison) to which they were originally allocated? | ++  +  −  NR  NA | Comments: |
| **4.3 Was the study sufficiently powered to detect an intervention effect (if one exists)?**  A power of 0.8 (that is, it is likely to see an effect of a given size if one exists, 80% of the time) is the conventionally accepted standard.  Is a power calculation presented? If not, what is the expected effect size? Is the sample size adequate? | ++  +  −  NR  NA | Comments: |
| **4.4 Were the estimates of effect size given or calculable?**  Were effect estimates (e.g. relative risks, absolute risks) given or possible to calculate? | ++  +  −  NR  NA | Comments: |
| **4.5 Were the analytical methods appropriate?**  Were important differences in follow-up time and likely confounders adjusted for?  If a cluster design, were analyses of sample size (and power), and effect size performed on clusters (and not individuals)?  Were subgroup analyses pre-specified? | ++  +  −  NR  NA | Comments: |
| **4.6 Was the precision of intervention effects given or calculable? Were they meaningful?**  Were confidence intervals or p values for effect estimates given or possible to calculate?  Were CI's wide or were they sufficiently precise to aid decision-making? If precision is lacking, is this because the study is under-powered? | ++  +  −  NR  NA | Comments: |
| **Section 5: Summary** | | |
| **5.1 Are the study results internally valid (i.e. unbiased)?**  How well did the study minimise sources of bias (i.e. adjusting for potential confounders)?  Were there significant flaws in the study design? | ++  +  − | Comments: |
| **5.2 Are the findings generalisable to the source population (i.e. externally valid)?**  Are there sufficient details given about the study to determine if the findings are generalisable to the source population? Consider: participants, interventions and comparisons, outcomes, resource and policy implications. | ++  +  − | Comments: |

Each study was awarded an overall Quality Rating: 1) ++ All or most of the checklist criteria were fulfilled, where they have not been fulfilled the conclusions are very unlikely to alter; 2) + Some of the checklist criteria were fulfilled, where they have not been fulfilled, or not adequately described, the conclusions are unlikely to alter; and 3) Few or no checklist criteria were fulfilled and the conclusions are likely or very likely to alter.As only one Phase IV study (DOLAM) with a 48-week follow-up was obtained from the SLR, meta-analysis could be performed. Thus, we proposed an additional analysis pooling the 4 studies extracted from the SLR (Phase III [n=3] and IV [n=1]).

1. **Risk of bias assessment**

A specific risk-of-bias analysis was performed with the Cochrane risk-of-bias tool for randomized trials (RoB 2) in publications proposed to be included in the meta-analysis. This tool assesses the methodological quality of studies included in systematic reviews and meta-analyses. The assessment is focused on 5 domains:

- D1: Bias arising from the randomization process.
- D2: Bias due to deviations from intended interventions.
- D3: Bias due to missing outcome data.
- D4: Bias in measurement of the outcome.
- D5: Bias in the selection of the reported result.

The risk of bias in the studies included in the SLR was assessed with the Cochrane risk-of-bias tool for randomized trials (RoB 2). This tool assesses the methodological quality of studies included in systematic reviews and meta-analyses. It aims to provide a systematic and transparent assessment of the internal validity of studies, thus helping reviewers to interpret the results more reliably. RoB 2 is structured into 5 domains. Each domain contains several questions to help the reviewer conclude the bias assessment (Table 6). As a result, we had the overall risk of bias and in each domain. The overall risk of bias could correspond to the worst risk in any of the domains. If a study had “some concerns” about the risk of bias in several domains, it was judged as at high risk of bias overall.

**Table 5.** RoB 2: Cochrane risk-of-bias tool for randomized trials

| Bias domain and signalling question* | Response options | | |
| --- | --- | --- | --- |
|  | **Lower risk** | **Higher risk** | **Other** |
| Bias arising from the randomization process |  |  |  |
| 1.1 Was the allocation sequence random? | Y/PY | N/PN | NI |
| 1.2 Was the allocation sequence concealed until participants were enrolled and assigned to interventions? | Y/PY | N/PN | NI |
| 1.3 Did baseline differences between intervention groups suggest a problem with the randomization process? | N/PN | Y/PY | NI |
| Risk-of-bias judgment (low/high/some concerns) |  |  |  |
| Optional: What is the predicted direction of bias arising from the randomization process? | | | |
| Bias due to deviations from intended interventions |  |  |  |
| 2.1 Were participants aware of their assigned intervention during the trial? N/PN Y/PY NI | N/PN | Y/PY | NI |
| 2.2 Were carers and people delivering the interventions aware of participants' assigned intervention during the trial? N/PN Y/PY NI | N/PN | Y/PY | NI |
| 2.3 If Y/PY/NI to 2.1 or 2.2: Were there deviations from the intended intervention that arose because of the trial context? N/PN Y/PY NA/NI | N/PN | Y/PY | NI |
| 2.4 If Y/PY/NI to 2.3: Were these deviations likely to have affected the outcome? | N/PN | Y/PY | NI |
| 2.5 If Y/PY to 2.4: Were these deviations from intended intervention balanced between groups? | Y/PY | N/PN | NI |
| 2.6 Was an appropriate analysis used to estimate the effect of assignment to intervention? | Y/PY | N/PN | NI |
| 2.7 If N/PN/NI to 2.6: Was there potential for a substantial impact (on the result) of the failure to analyze participants in the group to which they were randomized? | N/PN | Y/PY | NI |
| Risk-of-bias judgment (low/high/some concerns) |  |  |  |
| Optional: What is the predicted direction of bias due to deviations from intended interventions? | | | |
| Bias due to missing outcome data |  |  |  |
| 3.1 Were data for this outcome available for all, or nearly all, participants randomized? | Y/PY | N/PN | NI |
| 3.2 If N/PN/NI to 3.1: Is there evidence that the result was not biased by missing outcome data? | Y/PY | N/PN | NI |
| 3.3 If N/PN to 3.2: Could missingness in the outcome depend on its true value? | N/PN | Y/PY | NI |
| 3.4 If Y/PY/NI to 3.3: Is it likely that missingness in the outcome depended on its true value? | N/PN | Y/PY | NI |
| Risk-of-bias judgment (low/high/some concerns) |  |  |  |
| Optional: What is the predicted direction of bias due to missing outcome data? |  |  |  |
| Bias in measurement of the outcome |  |  |  |
| 4.1 Was the method of measuring the outcome inappropriate? | N/PN | Y/PY | NI |
| 4.2 Could measurement or ascertainment of the outcome have differed between intervention groups? | N/PN | Y/PY | NI |
| 4.3 If N/PN/NI to 4.1 and 4.2: Were outcome assessors aware of the intervention received by study participants? | N/PN | Y/PY | NI |
| 4.4 If Y/PY/NI to 4.3: Could assessment of the outcome have been influenced by knowledge of intervention received? | N/PN | Y/PY | NI |
| 4.5 If Y/PY/NI to 4.4: Is it likely that assessment of the outcome was influenced by knowledge of intervention received? | N/PN | Y/PY | NI |
| Risk-of-bias judgment (low/high/some concerns) |  |  |  |
| Optional: What is the predicted direction of bias in measurement of the outcome? |  |  |  |
| Bias in selection of the reported result |  |  |  |
| 5.1 Were the data that produced this result analyzed in accordance with a prespecified analysis plan that was finalized before unblinded outcome data were available for analysis? | Y/PY | N/PN | NI |
| Is the numerical result being assessed likely to have been selected, on the basis of the results, from: | | | |
| 5.2 ... multiple eligible outcome measurements (eg, scales, definitions, time points) within the outcome domain? | N/PN | Y/PY | NI |
| 5.3 ... multiple eligible analyses of the data? | N/PN | Y/PY | NI |
| Risk-of-bias judgment (low/high/some concerns) |  |  |  |
| Optional: What is the predicted direction bias due to selection of the reported results? | | | |
| Overall bias |  |  |  |
| Risk-of-bias judgment (low/high/some concerns) |  |  |  |
| Optional: What is the overall predicted direction of bias for this outcome? |  |  |  |

Y=yes; PY=probably yes; PN=probably no; N=no; NA=not applicable; NI=no information. *Signalling questions for bias due to deviations from intended interventions relate to the effect of assignment to intervention
