# Supplementary material for: Is less truly more? – reassessing antiretroviral efficacy – a safety analysis for HIV patients switching from triple to double regimens with integrase inhibitors: A systematic review and meta-analysis
Source: Medicine (Baltimore). 2025 Oct 17;104(42):e45152. doi: 10.1097/MD.0000000000045152 (PMC12537199; doi:10.1097/MD.0000000000045152)
Supplement: Supplementary file 2 [file medi-104-e45152-s002.docx]

Supplemental Content. Table 1 Baseline regimens of late-switch studies.

|  | TANGO (48,96,144) ^†^ | | TANGO-196 | | SWORD-48 | | SWORD-100 | | SWORD-148 | |
| --- | --- | --- | --- | --- | --- | --- | --- | --- | --- | --- |
| Regimen | **3DR or 4DR**  **(TAF-BR)**  **(n = 372)** | **2DR**  **(DTG/3TC)**  **(n = 369)** | **2DR LS**  **(DTG/3TC)**  **(n = 298)** | **2DR ES**  **(DTG/3TC)**  **(n = 369)** | **3DR or 4DR**  **(CAR)**  **(n = 511)** | **2DR**  **(DTG/RPV)**  **(n = 513)** | **2DR LS**  **(DTG/RPV)**  **(n = 477)** | **2DR ES**  **(DTG/RPV)**  **(n = 513)** | **2DR LS**  **(DTG/RPV)**  **(n = 477)** | **2DR ES**  **(DTG/RPV)**  **(n = 513)** |
| INSTI | **296 (79.6)** | **289 (78.3)** | **242 (81)** | **289 (78.3)** | **97 (19)** | **105 (20)** | **89 (19)** | **105 (20)** | **(19)** | **(20)** |
| DTG | -- | -- | -- | -- | -- | -- | -- | -- | -- | -- |
| EVG/COBI | 249 (66.9) | 243 (65.9) | 202 (68) | 243 (65.9) | -- | -- | -- | -- | -- | -- |
| EVG | -- | -- | -- | -- | -- | -- | -- | -- | -- | -- |
| BIC | -- | -- | -- | -- | -- | -- | -- | -- | -- | -- |
| RAL | -- | -- | -- | -- | 4 (1) | 4(1) | 41 (9) | 43 (8) | -- | -- |
| NNRTI | **48 (12.9)** | **51 (13.8)** | **33 (11)** | **51 (13.8)** | **278 (54)** | **275 (54)** | **267 (56)** | **275 (54)** | **(56)** | **(54)** |
| RPV | 45 (12.1) | 43 (11.7) | 30 (10) | 43 (11.7) | -- | -- | -- | -- | -- | -- |
| EFV | -- | -- | -- | -- | 62 (12) | 62 (12) | 182 (38) | 185 (36) | -- | -- |
| NRTI | **372 (100)**^†^ | **369 (100)** ^†^ | -- | **--** | **--** | **--** | -- | -- | -- | -- |
| ABC/3TC | -- | -- | -- | -- | -- | -- | -- | -- | -- | -- |
| 3TC | -- | -- | -- | -- | -- | -- | -- | -- | -- | -- |
| TAF | -- | -- | -- | -- | -- | -- | -- | -- | -- | -- |
| TDF | -- | -- | -- | -- | **359 (70)** | **374 (73)** | -- | -- | -- | -- |
| FTC | -- | -- | -- | -- | **341 (67)** | **352 (69)** | -- | -- | -- | -- |
| PI | **28 (7.5)** | **29 (7.9)** | **23 (8)** | **29 (7.9)** | **136 (27)** | **133 (26)** | **121 (25)** | **133 (26)** | **(25)** | **(26)** |
| RTV or COBI- boosted darunavir | 27 (7.3) | 25 (6.8) | 22 (7) | 25 (6.8) | 6 (1) | 9 (2) | 35 (7) | 58 (11) | -- | 6 (1) |
| Nº of patients (%).^†^All patients were on a TAF-based regimen prior to randomization. DR *drug regimens*, ABC a*bacavir*, BIC: *bictegravir*, CAR *current* *antiretroviral* *regimens*, COBI *cobicistat*, DTG *dolutegravir*, EFV *efavirenz*, ES *early-switch* EVG *elvitegravir*, FTC *emtricitabine*, INSTI *integrase* *strand* *transfer* | | | | | | | | | | |
