# Supplementary material for: Is less truly more? – reassessing antiretroviral efficacy – a safety analysis for HIV patients switching from triple to double regimens with integrase inhibitors: A systematic review and meta-analysis
Source: Medicine (Baltimore). 2025 Oct 17;104(42):e45152. doi: 10.1097/MD.0000000000045152 (PMC12537199; doi:10.1097/MD.0000000000045152)
Supplement: Supplementary file 3 [file medi-104-e45152-s003.docx]

**
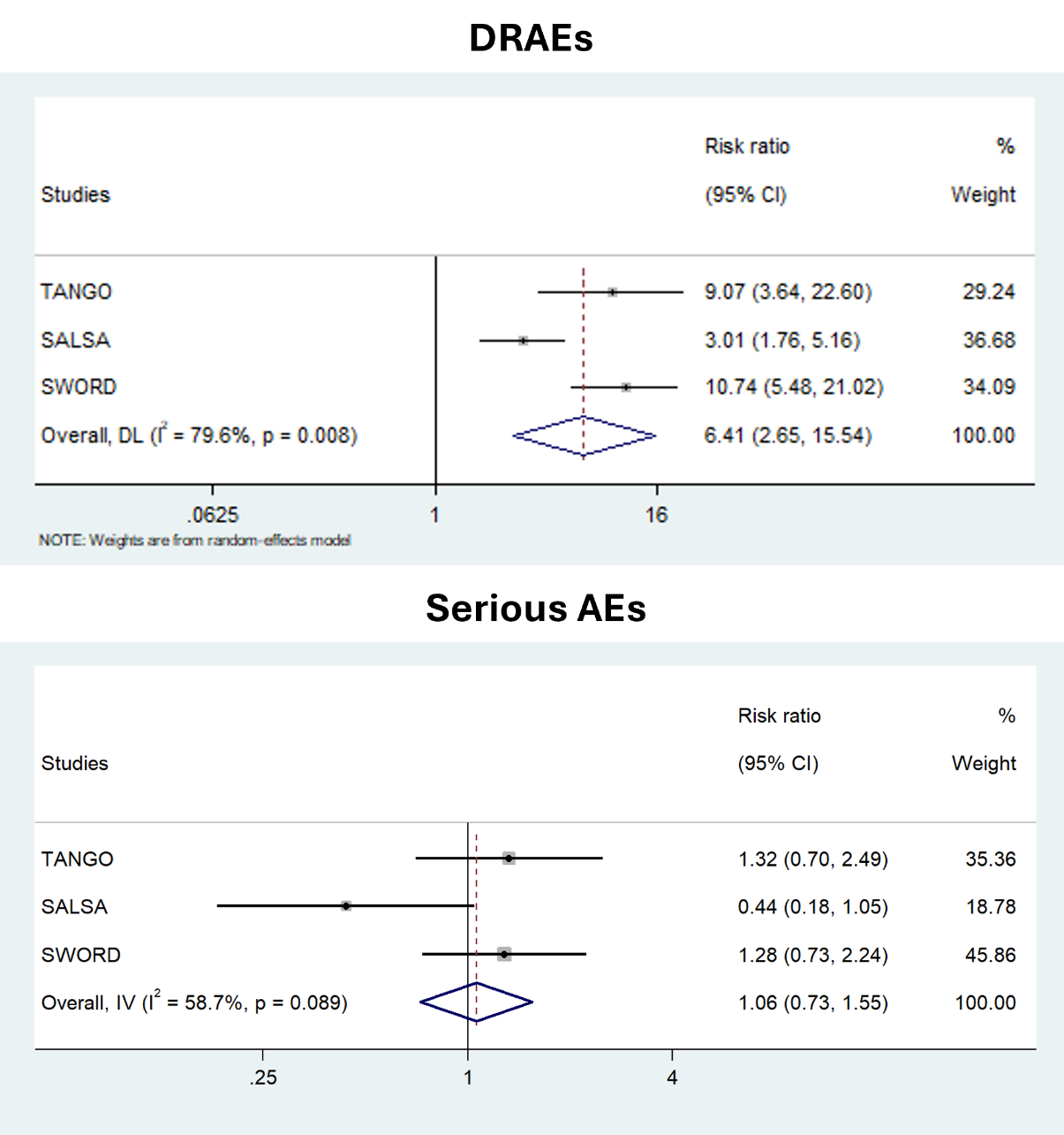
**

Supplementary Figure 1 Relative risks of developing DRAEs and Serious AEs excluding DOLAM study. Forest plots displaying the risk ratios of developing drug-related adverse events (top) and serious adverse events (bottom) and the relative weights of the three studies included in the meta-analysis. DRAEs *drug-related adverse events,* AEs *adverse* *events*, DL *DerSimonian-Laird estimate of tau²,* IV *inverse* *variance*, CI *confidence* *intervals,* I2 *heterogeneity index*. Patients included: 2258


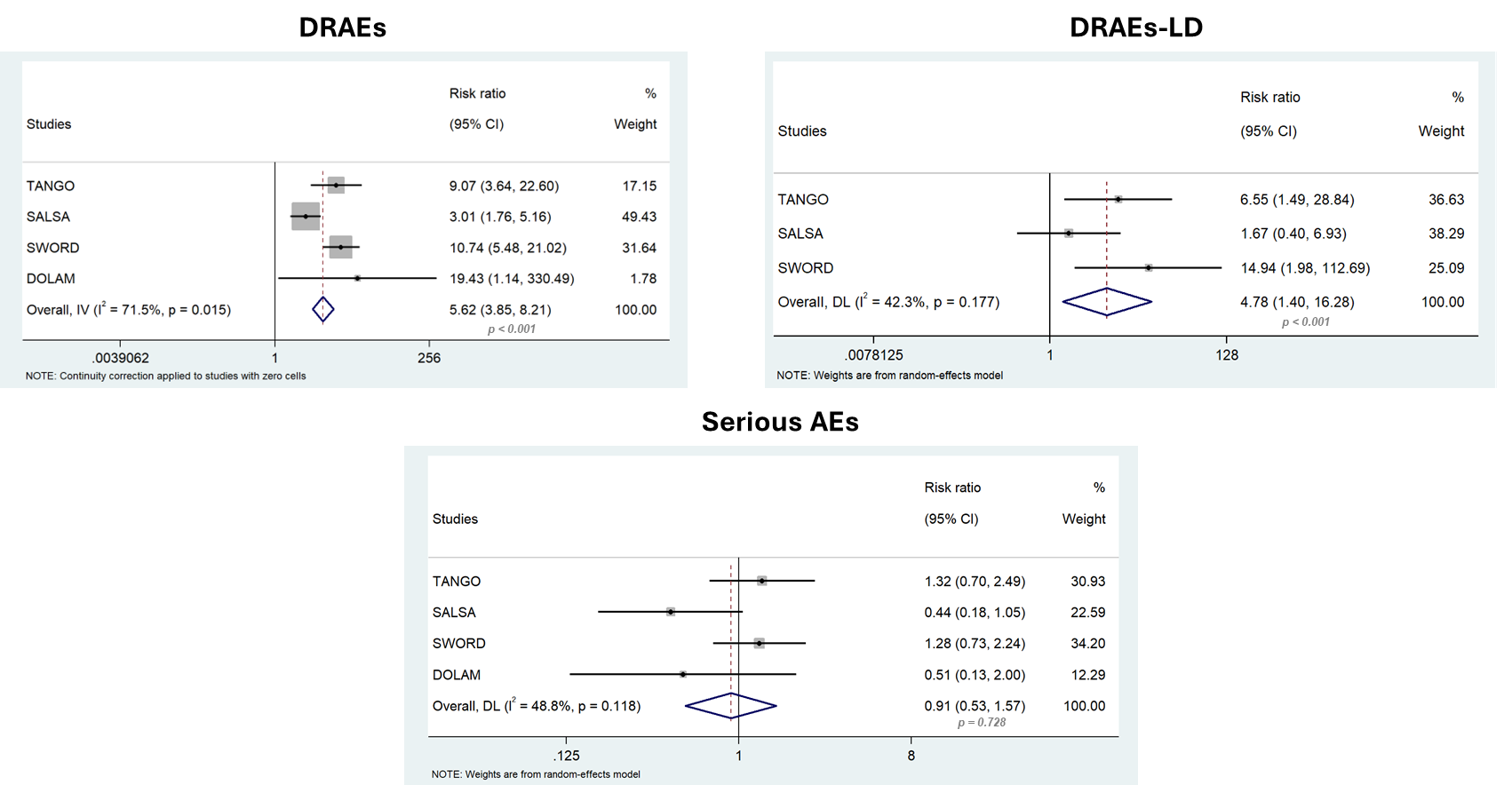


Supplementary Figure 2 Sensitivity Analysis. Forest plots displaying the sensitive analysis for the calculation of the risk ratios of developing drug-related adverse events (top-left), drug-related adverse events leading to discontinuation (top-right), and serious adverse events (bottom), and the relative weights of the studies included in the meta-analysis (four studies for drug-related adverse events and drug-related adverse events leading to discontinuation analyses and three studies for the sensitivity analysis of serious adverse events). DRAEs *drug-related adverse events,* DRAEs-LD *drug-related adverse events,* AEs *adverse* *events*, DL *DerSimonian-Laird estimate of tau²,* IV *inverse* *variance*, CI *confidence* *intervals,* I^2^ *heterogeneity index*. Patients included: 2523 for DRAEs, 2258 for DRAEs-LD and 2258 for serious AEs.
